# Multiplexed, rapid detection of H5N1 using a PCR-free nanoparticle-based genomic microarray assay

**DOI:** 10.1186/1472-6750-10-74

**Published:** 2010-10-13

**Authors:** Jiangqin Zhao, Shixing Tang, James Storhoff, Sudhakar Marla, Y Paul Bao, Xue Wang, Eric Y Wong, Viswanath Ragupathy, Zhiping Ye, Indira K Hewlett

**Affiliations:** 1 Lab of Molecular Virology, Center for Biologics Evaluation and Research, Food and Drug Administration, Bethesda, MD 20892, USA; 2 Nanosphere Inc., 4088 Commercial Avenue, Northbrook, IL 60062, USA; 3 Division of Viral Products, Center for Biologics Evaluation and Research, Food and Drug Administration, 1401 Rockville Pike, Rockville, MD 20852, USA

## Abstract

**Background:**

For more than a decade there has been increasing interest in the use of nanotechnology and microarray platforms for diagnostic applications. In this report, we describe a rapid and simple gold nanoparticle (NP)-based genomic microarray assay for specific identification of avian influenza virus H5N1 and its discrimination from other major influenza A virus strains (H1N1, H3N2).

**Results:**

Capture and intermediate oligonucleotides were designed based on the consensus sequences of the matrix (M) gene of H1N1, H3N2 and H5N1 viruses, and sequences specific for the hemaglutinin (HA) and neuraminidase (NA) genes of the H5N1 virus. Viral RNA was detected within 2.5 hours using capture-target-intermediate oligonucleotide hybridization and gold NP-mediated silver staining in the absence of RNA fragmentation, target amplification, and enzymatic reactions. The lower limit of detection (LOD) of the assay was less than 100 fM for purified PCR fragments and 10^3 ^TCID_50 _units for H5N1 viral RNA.

**Conclusions:**

The NP-based microarray assay was able to detect and distinguish H5N1 sequences from those of major influenza A viruses (H1N1, H3N2). The new method described here may be useful for simultaneous detection and subtyping of major influenza A viruses.

## Background

Influenza A virus consists of eight negative single-stranded RNA segments and can be classified into various subtypes based on antigenic differences of two surface glycoproteins: hemagglutinin (HA) and neuraminidase (NA). A total of 16 HA subtypes (H1-H16) and 9 NA subtypes (N1-N9) have been identified. Many subtypes of influenza virus are found in aquatic birds, and some of them have been reported to infect humans [1-5]. The two most common subtypes of influenza A virus currently circulating in humans are H1N1 and H3N2. The recent pandemic influenza virus, a novel swine influenza A/H1N1 virus (2009 A(H1N1)), which was first isolated in Mexico City in March 2009 [6], has spread to all states, and resulted in 9,079 hospitalized cases and 593 deaths in the United States as of September 11, 2009. In addition, highly pathogenic avian influenza (HPAI) virus H5N1 has spread throughout Asia, Europe, the Middle East and the African continent, and was also documented to infect humans [3,7]. H5N1 virus infection continues to progress with expanding host range and poses a serious global threat [8]. Rapid and sensitive diagnostic tools for the identification of influenza viruses are crucial for early detection, appropriate treatment, epidemiologic investigations, and timely responses to a pandemic threat.

The most common methods for identification of influenza A viruses mainly depend on virus isolation, culture, characterization by polymerase chain reaction (PCR) and hemagglutinin inhibition immunoassays, and require 3-7 days [8-11]. Nucleic acid testing (NAT) of influenza viruses has been widely implemented over the last decade using target amplification methods such as reverse transcription-PCR (RT-PCR), real-time RT-PCR, nucleic acid sequence-based amplification and loop-mediated isothermal amplification [12-15]. These technologies are being employed for the rapid diagnosis of influenza A, in particular the subtyping of the H5 gene [14,16-19]. For example, the Taqman influenza A/H5 Virus Detection Kit (Applied Biosystems, Foster City, CA), a PCR-based method, was developed as an essential research and diagnostic tool for detection of a broad range of subtypes of influenza viruses [20]. Additionally, Hoffmann et al. (2007) described a rapid real-time RT-PCR test specific for the HPAI virus H5N1 (Qinghai clade 2.2) without the need for sequencing [21]. These methods use universal primers and probes to detect all subtypes of influenza A, and specific primers and probes to distinguish HA and NA gene subtypes in a simple, single tube assay format following RNA isolation [13,14,18].

Microarray test methods have proven to be powerful tools for viral identification and subtyping [2,22-25]. For example, the FluChip microarray has been reported to detect H1N1, H3N2 and H5N1 strains in less than 12 hours [22,26]. The MChip microarray was able to identify influenza A virus with 95% sensitivity and 92% specificity [23,27]. CombiMatrix Corporation has also developed a semiconductor-based Influenza A Research Microarray that can detect all known subtypes of influenza A viruses within 5 hours [28]. More recently, a low-density microarray utilizing the NanoChip400 system (Nanogen Inc), which employs one probe for the conserved M gene and 97 probes for the cleavage site region of HA gene, was described to be a useful diagnostic tool for H5N1 virus [29,30]. However, all of these microarray based assays require two or more enzymatic amplification steps of influenza viral RNA prior to hybridization. In addition, detection requires labeling of multiple probes or incorporation of fluorescent dye- or biotin-conjugated nucleotides into double-stranded DNA (dsDNA) generated by RT-PCR. Moreover, the sensitivity of the conventional microarray assay relies on the efficiency of target amplification and hybridization of amplicons and probes. The multiple steps involved in these assays make them complicated, expensive, time consuming, susceptible to contamination, and may produce false negative results due to the presence of gene mutations, PCR inhibitors and RNA degradation. The design of multiple, specific primer sets and assay optimization pose major challenges [22].

In recent years there has been increasing interest in the use of nanoparticles (NP) coupled with silver staining for diagnostic applications [31-33] due to the higher sensitivities achievable by this approach compared with the fluorescent dyes that are commonly used in microarray assays. This modified microarray system allows direct detection of single nucleotide polymorphisms (SNPs) in human genomic DNA samples without the need for template amplification [33]. In this report, we describe the development of a new NP-based genomic microarray assay that specifically identifies H5N1 viral nucleic acid and simultaneously provides subtype identification of influenza A virus in the absence of target amplification procedures such as RT-PCR. The genomic microarray system has a high degree of hybridization efficiency and assay specificity. The method is also simple and rapid since the H5N1 viral genomic RNA is added directly to the slides for hybridization and can be detected using gold NP probes.

## Results

### Probes for the NP-based microarray assay

**Figure 1:**
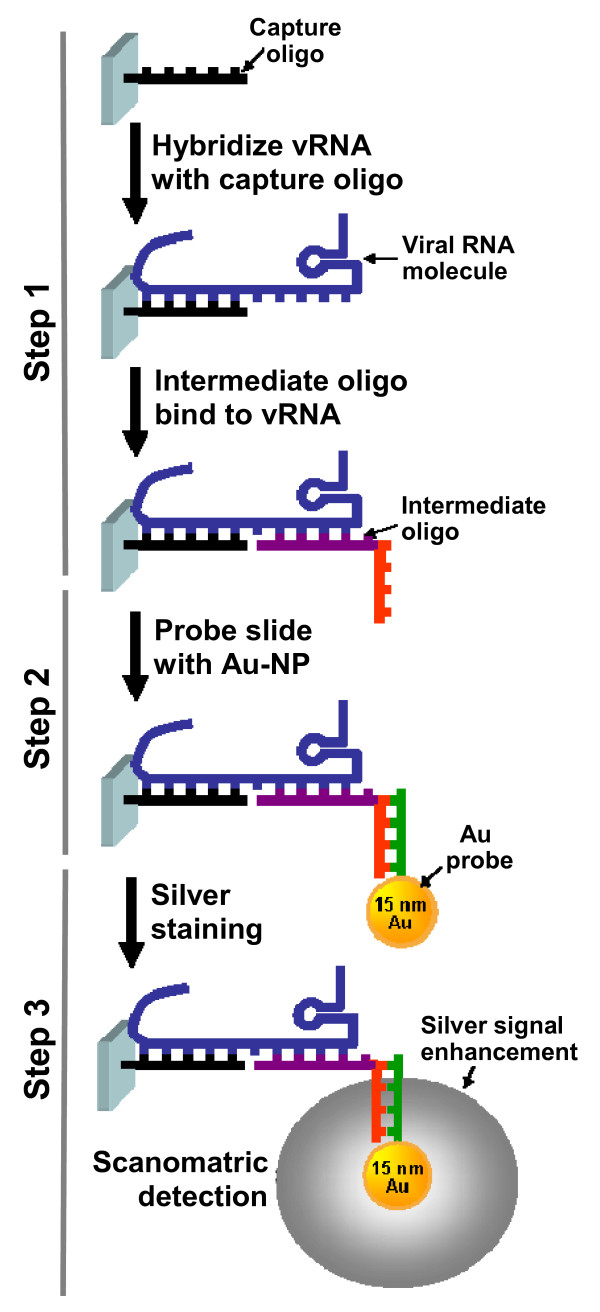
**Simplified schematic overview of the gold nanoparticle (NP)-based microarray assay**. The target is whole viral genome RNA (blue line), end modified capture oligos are 40 bp in length (black line) printed on the amino-modified glass slide and intermediate oligos are 65 bp in length (purple line) including 25-mer poly (A) tail (red line). The probe is 15 nm gold nanoparticles (NP) functionalized with poly dT tail (green line). The specific hybridizations between the capture oligo, RNA (or DNA) and intermediate oligo take place to form sandwich complexes in Step 1, then the gold NP probes bind to the complexes in Step 2, and silver staining is in Step 3.

Table 1 lists all capture and intermediate olignucleotide (oligo) probes designed for this study. Three degenerate capture oligos (M01, M02 and M03) were designed to bind to the consensus M gene sequence region of influenza A virus. An additional four capture oligos were designed to specifically bind to sequences from the H5 and N1 genes, respectively. The 11 specific capture oligos and a positive control oligo were synthesized and printed on the array. For each gene, four or five intermediate oligos that bind to a region adjacent to the capture sequences were designed and synthesized (refer to Fig. 1 for assay scheme).

**Table 1:** Capture, intermediate and PCR oligos sequences

Oligo's Name	Target gene	design purpose	sequences (5' to 3')
M01**	M	capture	CAGGTAGATRTTGAAAGATGAGYCTTCTAACCGAGGTCGA
M02**	M	capture	TGACTCCCAGCAYMRGTCTCATAGGCAAATGGTGACAACA
M03**	M	capture	AAGGCTATGGAGCARATGGCDGGATCRAGTGAGCARGCAG
H501	H5	capture	TCATCAATGTGCCGGAATGGTCTTACATAGTGGAGAAGGC
H502	H5	capture	CATACCCAACAATAAAGAGGAGCTACAATAATACCAACCA
H503	H5	capture	TAGAGGGAGGATGGCAGGGAATGGTAGATGGTTGGTATGG
H504	H5	capture	TTCCTAGATGTCTGGACTTATAATGCTGAACTTCTGGTTC
N101**	N1	capture	CAGYAAGGACAACAGTATAAGGATCGGTTCCARGGGGGAT
N102	N1	capture	CATTAATGAGTTGTCCTGTGGGTGAGGCTCCCTCCCCATA
N103**	N1	capture	CCTAATTATCACTATGAGGARTGCTCCTGTTATCCTGATG
N104**	N1	capture	TGCATAAGACCTTGTTTCTGGGTTGAGTTRATCAGAGGGC
PCtrl1		capture	ACTGTTTGTTATCTTGTTATCGTTATCTGA
Ipm01	M	intermediate	AAAGCCGAGATCGCACAGAGACTTGAAGATGTCTTTGCAG*
Ipm02	M	intermediate	AGCTCCAGTGCTGGTCTGAAAAATGATCTTCTTGAAAATT*
Ipm03	M	intermediate	GCCAAAGTCTATGAGGGAAGAATATCGAAAGGAACAGCAG*
Ipm04	M	intermediate	AATGGGGGTGCAGATGCAACGGTTCAAGTGATCCTCTCAC*
Ipm05	M	intermediate	CCACTAATCAGACATGAGAACAGAATGGTTTTAGCCAGCA*
Ipm06	H5	intermediate	GACTATGAAGAATTGAAACACCTATTGAGCAGAATAAACC*
Ipm07	H5	intermediate	TCCGTTGGGACATCAACACTAAACCAGAGATTGGTACCAA*
Ipm08	H5	intermediate	AGGCAATAGATGGAGTCACCAATAAGGTCAACTCGATCAT*
Ipm09	H5	intermediate	CATGACTCAAATGTCAAGAACCTTTACGACAAGGTCCGAC*
Ipm10	H5	intermediate	ACTGGCAATCATGGTAGCTGGTCTATCCTTATGGATGTGC*
Ipn11	N1	intermediate	AGAGAGCCGTTCATCTCATGCTCCCACTTGGAATGCAGAA*
Ipn12	N1	intermediate	GAGTCTGTTGCTTGGTCRGCAAGTGCTTGCCATGATGGCA*
Ipn13	N1	intermediate	TGTGCATGTGTAAATGGCTCTTGCTTTACTGTAATGACTG*
Ipn14	N1	intermediate	AATCACATGTGTGTGCAGGGATAATTGGCATGGCTCAAAT*
Ipn15	N1	intermediate	TCTTGGCCAGACGGTGCTGAGTTGCCATTCACCATTGACA*
^a^PCrl2		control probe	TCAGATAACGATAACAAGATAACAAACAGT
^b^SZAM-	M	PCR	AGTAGAAACAAGGTAGTTTTTT
MF	M	PCR	TAGATATTGAAAGATGAGTC
HA5F	H5	PCR	GGTATAATCTGTCAAAATGGAGA
HA5R	H5	PCR	TAACTACAATCTGAACTCACAAAT
NA6F	N1	PCR	TCCAAATCAGAAGATAATAACCAT
NA6R	N1	PCR	GAATGGCAACTCAGCACCGTCT

* 25-mer poly (A) tail added at 3' end of each intermediate oligonucleotides

** M gene degenerated capture oligos from H1N1, H3N2 and H5N1.

^a ^Gold NP was functionalized by PCrl2 oligo as a internal control probe.

^b^Sequence from publication by Zou (1997) [36].

### Specificity and sensitivity of the genomic microarray assay

A typical NP-based genomic microarray assay is performed by directly hybridizing purified viral RNA to the capture oligos attached to the slide and then introducing intermediate oligos with a poly-A tail to form a sandwich complex (Fig. 1, Step 1). The complex is then incubated with a poly dT-modified gold-NP probe that can bind to the intermediate oligos and be stained with silver solution (Steps 2 and 3). The enhanced light-scattering signal produced by the silver shell is detected by Nanosphere Verigene ^® ^reader.

**Figure 2:**
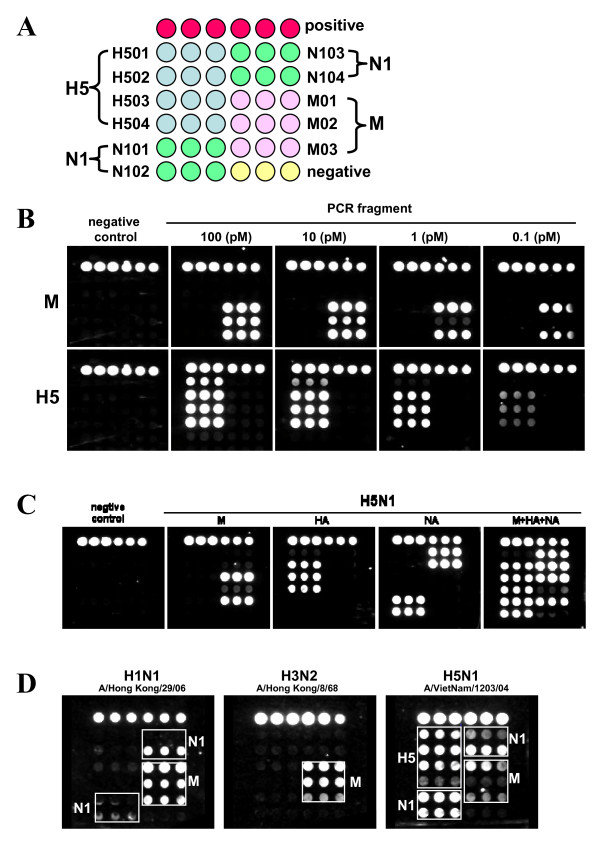
**Image from a Verigene ID™ detection system**. (A). Microarray layout with positive control capture (closed red circles on the top), negative control which uses printing buffer as capture (closed yellow circles), M, H5, and N1 gene captures (filled as variable color of closed circles, spotted in triplicate) are indicated. (B). The light shades represent strong silver staining signals. A portion of the microarray images for DNA oligonucleotide following hybridization with PCR fragment are shown and light shades represent greater silver intensities for the M and H5 genes. The spots at the top of each array are positive controls. (C). Silver staining image for individual or multiple PCR products are shown and labeled. (D). Typical genomic microarray silver staining image from 0.2 μg of H1N1, H3N2 or H5N1 viral RNA are shown. The boxed areas highlight hits for specific subtypes.

The detection sensitivity of the NP-based microarray assay was first evaluated using M and H5 gene PCR amplicons generated from the H5N1 virus strain 'A/Vietnam/1203/04' (Fig. 2B). A dose-response model in the assay signal intensity was observed when the PCR products were diluted from 100 pM to 100 fM, although different patterns resulted between the M and H5 genes, and among various capture oligos of the M gene. The M gene capture oligos M01 and M03 could detect at least 100 fM of PCR amplicon (S/CO = 6.2 ± 0.34 at 100 fM for capture M01) while three of the four capture oligos of the H5 gene also detected 100 fM of the H5 gene PCR amplicon (S/CO >1 at 100 fM for capture H502-H504). A third M gene capture oligo, M02, and the H5 gene capture oligo, H501, could only detect 1 pM of the target. It is noteworthy that the sensitivity of the assay is based on measurement of a purified PCR amplicon which may be different from genomic viral RNA (see below).

The PCR fragments of the M, H5 and N1 genes of H5N1 virus (A/Vietnam/1203/04) were hybridized separately or simultaneously to further evaluate the assay specificity (Fig. 2C). Specific signal was only generated in the areas printed with the corresponding gene-specific capture oligos (Fig. 2C, panel M, HA and NA). No interference was observed when multiple targets and intermediate oligos were mixed and incubated simultaneously (Fig. 2C, panel M+HA+NA). Therefore, the use of a three-step capture and three-level hybridization procedure in the current assay proved to be specific for detection of M, H5 and N1 genes of H5N1.

### Direct detection of H5N1 viral RNA

The next set of experiments focused on directly detecting viral RNA in the absence of enzymatic target amplification using the gold NP-based microarray assay (Fig. 2D and Table 2). The consensus M gene oligos within the microarray assay could detect the M gene from all three of the major subtypes of influenza A viruses (H1N1, H3N2 and H5N1) tested in our study. In addition, the N1 gene was detected in both H1N1 (A/Hong Kong/29/06) and H5N1 (A/Vietnam/1203/04) viruses, and the H5 gene was only detected in the H5N1 virus. No H5 or N1 signals were observed for H3N2 virus (A/Hong Kong/8/68). The results of 17 influenza A viruses tested for the M, H5 and N1 genes showed in Table 2. As a proof-of-concept experiment, these results indicated that our assay conditions and format permit the detection of different strains of H5N1 virus and were able to discriminate between the major subtypes of influenza A viruses H1N1, H3N2 and H5N1.

**Table 2:** Results of genomic array detection of influenza A viruses with specific captures

		Specific Captures		
Isolates	Subtype	H5	N1	M
A/Hong Kong/29/06	H1N1	-	+	+
A/Singapore/63/04	H1N1	-	+	+
A/Puerto Rico/8/34	H1N1	-	+	+
A/New Caledonia/20/99	H1N1	-	+	+
A/Hong Kong/8/68	H3N2	-	-	+
A/Wisconsin/67/05	H3N2	-	-	+
A/Panama/2007/99	H3N2	-	-	+
A/duck/Laos/3295/06	H5N1	+	+	+
A/Vietnam/1203/04	H5N1	+	+	+
A/Hong Kong/491/97	H5N1	+	+	+
A/Vietnam/1194/04	H5N1	+	+	+
A/Anhui/01/05	H5N1	+	+	+
A/Turkey/1/05	H5N1	+	+	+
A/Indonesia/05/05	H5N1	+	+	+
A/Turkey/Turkey/1/2005	H5N1	+	+	+
A/(H5) control antigen*	H5N1	+	+	+
A/Duck/Laos-PR8/CBER-RG1	H5N1	+	+	+

*H5N1 strain obtained from CDC (VA2701, Inactivated H5N1)

Due to the lack of well-characterized standards for influenza A virus RNA, the detection sensitivity of the NP-based microarray assay was evaluated by using two different sources of viral RNA. First, the well-quantified RNA transcribed from H1N1 M gene plasmid was used in the experiments. The assay detected approximately 10^5 ^copies of transcribed RNA based on signals from the M gene capture oligos M01 and M02 (data not shown). Second, viral RNA extracted from the H5N1 strain A/Vietnam/1203/04 with quantified TCID_50 _units was tested. Without RT-PCR amplification, the assay detected 10^3 ^TCID_50 _units of viral RNA per reaction with the specific H5, N1, and M gene capture oligos based on a S/CO of >1 (Table 3). Statistical analysis indicated that capture oligo N104 had the highest detection sensitivity for the A/Vietnam/1203/04 (H5N1) strain. In a parallel experiment, the NP-based microarray assay showed 10^3 ^fold lower sensitivity compared with the Taqman assay (data not shown).

**Table 3:** Signal/cutoff ratios of the H5N1 captures relevant to virus titer

		Virus titer (Log10 TCID_50 _units/rxn)		
Captures	10^5^	10^4^	10^3^	10^2^
positive	42.7 ± 0.91	38.2 ± 2.11	40.2 ± 2.01	37.1 ± 2.12
H502 (H5)	38.9 ± 1.31	6.9 ± 0.30	1.3 ± 0.76	0.1 ± 0.11
N104 (N1)	43.6 ± 2.79	28.2 ± 0.07	4.8 ± 0.88	0.2 ± 0.16
M01 (M)	42.1 ± 2.60	6.0 ± 1.26	1.2 ± 0.41	0.1 ± 0.07

## Discussion

The conventional microarray assay requires target amplification by PCR and incorporation of a fluorescent dye- or biotin-conjugated nucleotide into PCR products prior to hybridization. These assays are both time- and labor-consuming and challenging to perform since multiple instruments are required for amplification and detection. Special precautions are needed to prevent carry-over contamination since detection of the resulting PCR amplicons is not performed in the same tube. In addition, RT-PCR may fail to amplify the target gene due to the highly variable sequences among influenza viruses [22], and multiple PCRs assays may be required to determine the subtypes of influenza A viruses. In this study, we developed a rapid, simple gold NP-based microarray assay for the simultaneous detection of M, H5 and N1 genes of influenza A virus H5N1 and for differentiation of H5N1 from H1N1 and H3N2 viruses. Our system combines specificity and capability for multiplexed detection on a simplified microarray platform, and may potentially be used in resource-limited areas for monitoring or screening of viral strains circulating during an influenza pandemic. The unique advantages of the new system include: 1) direct detection of viral RNA without involvement of target amplification or other enzymatic steps; 2) capture of a wide variety of strains by using multiple capture oligos that target different viral genes and sequences; 3) simultaneous detection and subtyping of major influenza A viruses; 4) a relatively simple, and rapid format. After RNA extraction, 20 samples can be tested in approximately 2.5 hours. It is faster and less labor-intensive than previously reported assays such as the FluChip [22,26], MChip [23,27] and NanoChip 400 [29,30], which usually require 5-12 hours for detection.

Preliminary data indicate that the assay is able to achieve a high degree of sensitivity without PCR amplification. One reason is that the new detection method utilizes gold NP-based silver enhancement, which provides higher sensitivity than the traditional fluorescent labels. Another possible reason for higher detection sensitivity is the use of multiple capture and intermediate oligos to detect a target in multiple regions, which significantly increases the binding of gold NPs and could overcome the diagnostic challenge posed by potential gene mutations. The assay may also be suitable for point-of-care settings with further modifications of the detection systems. Indeed, the experiments we performed showed that the microarray signals could be visualized by the naked eye, without the need for a device, when sufficiently high viral RNA copies are present in the samples.

The genomic microarray assay involves three steps with three levels of capture-target-intermediate oligo hybridization as indicated in Fig. 1. Our results demonstrate that selection of target specific capture sequences is critical for performance of this assay, and the use of multiple capture and intermediate oligos is critical for detecting a wide variety of strains with the desired sensitivity. Three degenerate capture oligos for M gene (M01, M02 and M03) were designed to identify the major influenza A viruses (H1N1, H3N2 and H5N1) in our assay since the M gene is relatively well-conserved among influenza A viruses. Due to genetic variation of different virus strains, the performance of the different capture oligos varied (Fig. 2C and 2D). For example, A/Vietnam/1203/04 (H5N1) strain hybridized well with the M01 and M03 capture oligos but not with M02 (Fig. 2B and 2C). The alignment of the three capture sequences with the M gene sequence of A/Vietnam/1203/04 strain indicated that there were two and four degenerate nucleotide substitutions in M01 and M03, respectively, but there were three degenerate nucleotide substitutions and eleven nucleotide mismatches in M02. This may explain why M02 was not able to detect the A/Vietnam/1203/04 strain but performed well with other strains, including H1N1 (Fig. 2D, A/Hong Kong/29/06), H3N2 (A/Hong Kong/8/68), and certain H5N1 strains. Furthermore, the same oligo design strategy was used for subtyping the H5 and N1 genes, and similar results were observed. For example, the N1 gene capture oligo N104 can consistently detect the majority of viral strains tested whereas other N1 gene capture oligos exhibit more variation in performance across strains. Nonetheless, capture oligo N102 performed better than N104 in some H5N1 strains. These results suggest that multiple captures are necessary for improving detection sensitivity.

Using this multiple capture approach, the presence of influenza A viral RNA was verified in all seventeen strains of different influenza A viruses tested. The H5 gene was correctly identified in well-characterized H5N1 samples and the N1 gene was correctly identified in all H5N1 and H1N1 samples tested. No cross-reactivity was observed when other influenza A strains were tested. These results demonstrate the specificity and accuracy of our assay for detecting and subtyping H5N1 strains. Our current NP-based genomic array could detect 10^3 ^TCID_50 _units of H5N1 virus per reaction, which is within the range of proposed detection limits for influenza diagnostics [34].

## Conclusions

A novel, PCR-free, NP-based genomic microarray assay for subtyping influenza A virus H5N1 was developed and evaluated using different influenza A strains. Preliminary data indicate that the method may be useful in surveillance and rapid identification of influenza A viral infection, particularly during H5N1 outbreaks. This proof-of-concept study provides evidence that the new system may be used to characterize multiple influenza A viruses during a pandemic and has the potential to simultaneously detect multiple, major influenza A viruses. We believe that assay sensitivity can be further improved through sample enrichment and optimization of assay conditions. Further studies are needed to validate the current method with additional H5N1 strains and clinical samples. The specific capture oligos for targeting H1, H3, H7, N2, and other genes in additional influenza A viruses will be generated in the future to expand the current identification panel. Thus, this new NP-based genomic array system provides the flexibility to be rapidly modified to detect new targets. Since the viral HA and NA genes of the recent outbreak of swine influenza 2009 A(H1N1) are 27.2% and 18.2% distinct respectively, in amino acid sequence, from the 2008 H1N1 strain [35], it is possible to modify this new NP-based genomic microarray using specific oligos for the 2009 influenza A (H1N1) virus or other emerging pandemic influenza strains to facilitate identification and differentiation of these viruses from other circulating influenza A viral strains.

## Methods

### Samples and viral RNA extraction

Influenza virus isolates A/Hong Kong/29/06(H1N1), A/Singapore/63/04 (H1N1), A/Hong Kong/8/68(H3N2), A/Wisconsin/67/05(H3N2), H5N1 viruses (A/duck/Laos/3295/06; A/Vietnam/1203/04; A/Hong Kong/491/97; A/Vietnam/1194/04; A/Anhui/01/05; A/Turkey/1/05 and A/Indonesia/05/05), were cultured in embryonated chicken eggs in CBER, FDA. The plasmids pHW-pr8M (H1N1), pCR-II Topo HA (H5N1) and pCR-II Topo NA (H5N1) for HA, NA and M genes were provided by CDC (CDC, Atlanta, GA), some influenza virus strains were purchased from ZeptoMetrix (ZeptoMetrix corp., NY). Viral RNA of human immunodeficiency virus type one (HIV-1) and West Nile virus (WNV) (isolate NY 2001-6263) were cultured in-house and served as negative controls. Genomic viral RNA was extracted directly from allantoic fluid or cell culture supernatant with QIAamp Viral RNA Mini Kit (QIAGEN, Valencia, CA). The purified RNA was quantified using NanoDrop (NanoDrop Technologies, Inc. DE).

### Primer design and RT-PCR

The PCR primers for M, H5 and N1 genes of H5N1 were designed and listed in Table 1[36]. H5N1 viral RNA (A/Vietnam/1203/04) was first transcribed into cDNA using SuperScript™ III First-Strand Synthesis System for RT-PCR (Invitrogen, CA). cDNA was used as template to perform a standard RT-PCR. The reaction mixture consisted of 15 μl of 2× PCR Master Mix (Promega Corp. WI), 25 pM of each forward and reverse primers, 2.0 μl of cDNA template. RNase free water was added to bring the total volume to 30 μl. Amplification involved an initial denaturation step at 95°C for 3 min, and 35 cycles of denaturation at 94°C for 30 sec, annealing at 50°C for 35 sec and extension at 72°C for 1.5 min, followed by a final step of extension at 72°C for 7 min. The PCR products were electrophoresed on 1.0% agarose gel in Tris-acetate-EDTA buffer. A 1.0 kb DNA ladder was used as a molecular weight marker. The position of bands at 1747 bp, 1335 bp and 1015 bp in agarose gel represented full length amplification products of H5, N1 and M genes, respectively.

### Capture and intermediate oligos

By using nucleotide sequences available in the Influenza Primer Design Resource (IPDR) (http://www.ipdr.mcw.edu/fludb/search), multiple sequence alignments of H5, N1 and M genes were performed using MEGA 4 [37] and Vector NTI Version 10 (Invitrogen, CA). Influenza A virus RNA segment 7 encodes two matrix proteins M1 and M2. The M1 protein is a highly conserved protein of 252-amino acids [38,39]. Multiple capture and intermediate oligos complementary to the conserved regions of M gene were selected from over 166 known sequences corresponding to different subtypes of influenza A viruses, including H1N1, H3N2 and H5N1, whereas H5 oligos were chosen from the conserved regions of over 40 known sequences of H5N1, and N1 oligos were selected from 42 known sequences for both H1N1 and H5N1. Both capture and intermediate oligos were also analyzed using the primer analysis software (http://www.operon.com/oligos/toolkit.php) and Primer Express 3.0 (Applied Biosystems, Foster City, CA) to ensure no significant interference when they were mixed and subjected to the same hybridization conditions. The capture oligos we designed were modified with 5'-Amino-C_6_-Modifier while a 25-mer poly (A) tail was added at the 3' end of intermediate oligos during the synthesis at IDT Inc. (Integrated DNA Technologies, IA). Selected capture/intermediate oligos were also screened by mfold software (http://mfold.bioinfo.rpi.edu/cgi-bin/rna-form1.cgi) [40] to prevent RNA secondary structure from inhibiting hybridization. Capture oligos which did not bind to any known sequences of influenza A viruses were included as array internal controls [31]. Detailed information about the sequences of capture and intermediate oligos used in this study is listed in Table 1.

### NP-based microarray assay

Target-specific capture oligo, positive-control oligo and printing buffer (used as the negative control) were arrayed onto CodeLink Activated slides (SurModics, Eden Prairie, MN) at Nanosphere Inc. (Northbrook, IL) [31]. Each slide contained 10 identical sub-arrays partitioned by a hybridization gasket, thus enabling 10 tests per slide. Aqueous DNA-modified gold NP probe solutions were prepared and supplied by Nanosphere Inc. 0.2-1.0 μg of viral RNA samples and 10 nM of the intermediate oligos (final concentration) were diluted in 100 μl final volume of the hybridization buffer containing 5 × SSC (Invitrogen, Carlsbad, CA), 0.05% sorbitan mono-9-octa decenoate poly(oxy-1,1-ethanediyl), 0.05% Tween-20 (Sigma) and 40% formamide (Sigma), and applied to the microarray. PCR amplicons were first denatured at 95°C for 5 min and cooled down on ice for 2 min before loading. The arrays were incubated for 90 min at 40°C with shaking at 500 rpm in a hybridization oven (Step 1, Fig. 1). After the incubation, the gasket was removed and slides soaked three times in wash buffer A [0.5 N NaNO_3_, 0.01% SDS and 0.05% Tween-20] and rinsed once in wash buffer B [0.5 N NaNO_3_] and then dried. The NP probe solution was incubated with the slide covered by a new hybridization chamber for 30 min at 40°C (Step 2). After washing with buffer B, the slides were stained with 2 ml of the mixture of the Silver Enhancer A and B solutions (Nanosphere Inc., Northbrook IL) for 7.5 min at room temperature (Step 3). The light-scattering signal produced by the silver-enhanced gold NPs was captured by a photosensor and converted to a TIFF image by using a Verigene Reader (V1.1.6, Nanosphere, Inc.). The resulting TIFF images were analyzed using GenePix 6 software (Molecular Devices).

### Determination of sensitivity and specificity

A serial dilution of the quantitated M gene and H5 gene PCR amplicons (ranging from 100 to 0.1 pM) was analyzed using the NP-based microarray assay to determine the limit of detection (LOD). The LOD was defined as the lowest concentration at which a positive signal could be detected. PCR fragments for H5, N1 and M genes from the H5N1 strain (A/Vietnam/1203/04) were loaded in the sub-array separately and simultaneously to determine whether any cross-hybridization occurred. Specificity of the multiplexed microarray assays was evaluated by testing cross-reactivity with RNA extracted from three subtypes of influenza A virus and other viral pathogens such as HIV-1 and WNV.

### Data analysis

For images collected by Verigene Reader, background noise was subtracted from the raw images using GenePix 6.0 data analysis software. The cutoff value was the sum of the means of the pixel intensity of three negative controls plus 3 standard deviations (SD). Samples with signal-to-cutoff (S/CO) ratios equal to or greater than 1.00 were considered positive.

## Abbreviations

HA: Hemagglutinin; NA: Neuraminidase; NAT: Nucleic Acid Testing; NP: Nanoparticle; LOD: Limit of Detection; HPAI: High Pathogenic Avian Influenza; WNV: West Nile Virus; TCID_50_: 50% of Tissue Culture Infective Dose;

## Competing interests

The authors declare that they have no competing interests.

## Authors' contributions

JZ designed oligo and carried out the experimental study, contributed to the analysis of the results and drafted the manuscript. SM, YPB, XW, EYW, VR and ZY participated in the analysis of the results and drafting of the manuscript. ST, JS and IKH conceived of the study, and participated in its design and coordination and helped to draft the manuscript. All authors contributed in the preparation of the manuscript and approved the final manuscript.
